# CCN1/Cyr61 associates with β-amyloid levels in human cerebrospinal fluids

**DOI:** 10.1016/j.isci.2026.116244

**Published:** 2026-06-05

**Authors:** Mitsuru Shinohara, Hiroyuki Momota, Tsuyoshi Saito, Chisako Takenobu, Kensaku Kasuga, Ghupurjan Gheni, Kaori Kawai, Maho Morishima, Yuko Saito, Akihiro Shindo, Fumihiko Yasuno, Takeshi Ikeuchi, Akio Fukumori, Naoyuki Sato

**Affiliations:** 1 Department of Aging Neurobiology, Center for Development of Advanced Medicine for Dementia, National Center for Geriatrics and Gerontology, 7-430, Morioka, Obu, Aichi 474-8511, Japan; 2 Department of Aging Neurobiology, Graduate School of Medicine, The University of Osaka, 2-2, Yamadaoka, Suita, Osaka 565-0871, Japan; 3 Department of Neurosurgery, National Center for Geriatrics and Gerontology, 7-430, Morioka, Obu, Aichi 474-8511, Japan; 4 Department of Diagnostic Innovation Science, Center for Development of Advanced Medicine for Dementia, National Center for Geriatrics and Gerontology, 7-430, Morioka, Obu, Aichi 474-8511, Japan; 5 Department of Neuropathology (Brain Bank for Aging Research), Tokyo Metropolitan Institute for Geriatrics and Gerontology, 35-2, Sakae-machi, Itabashi-ku, Tokyo 173-0015, Japan; 6 Department of Neurology, Graduate School of Medicine, Mie University, 1577 Kurima Machiyacho, Tsu City, Mie 514-8507, Japan; 7 Department of Psychiatry, National Center for Geriatrics and Gerontology, Obu, Aichi, Japan; 8 Department of Molecular Genetics, Brain Research Institute, Niigata University, Chuo-ku, Niigata, Japan; 9 Department of Pharmacotherapeutics II, Faculty of Pharmacy, Osaka Medical and Pharmaceutical University, 4-20-1 Nasahara, Takatsuki, Osaka 569-1094, Japan; 10 Department of Neuropsychiatry, Graduate School of Medicine, The University of Osaka, Suita, Japan; 11 Department of Mental Health Promotion, Graduate School of Medicine, The University of Osaka, Toyonaka, Japan

**Keywords:** neurology, diagnostics, clinical neuroscience

## Abstract

CCN1, also called Cyr61, is a secreted protein involved in diverse biological processes including senescence. While elevated in brains of Alzheimer’s disease (AD) models and patients, CCN1/Cyr61 levels in cerebrospinal fluid (CSF) remain unclear. Using a high-sensitive quantification method, we analyzed CSF samples from 79 subjects (age: 77.7 ± 5.4 years [63–94], MMSE: 20.1 ± 5.9 [0–30]) who underwent CSF tap test. CCN1/Cyr61 showed strong associations with Aβ40 (r = 0.67), Aβ42 (r = 0.48), Aβ42/40 ratio (r = −0.53), and phospho-tau levels (r = 0.53) (all; *p* < 0.0001). CCN1/Cyr61 also moderately associated with glial markers, YKL-40, sTREM2, CD163, and a lymphatic endothelial marker, LYVE-1 (r ≈ 0.4). While these cellular markers also associated with Aβ and phospho-tau, effects were much weaker. Collectively, CCN1/Cyr61 is associated with Aβ species and p-tau in CSF. These associations are considerably stronger than those observed with typical glial or other cellular markers, providing a clue to understanding the link between senescence and AD pathology at the levels of fluid biomarkers.

## Highlights

• We developed a highly sensitive CCN1/Cyr61 quantification assay using SIMOA
• CCN1/Cyr61 in CSF strongly correlated with Aβ species and phosphorylated tau
• CCN1/Cyr61 showed moderate associations with glial and other cellular markers
• These findings indicate CCN1/Cyr61 may be involved in Alzheimer’s disease pathology

## Graphical abstract

**Figure:**
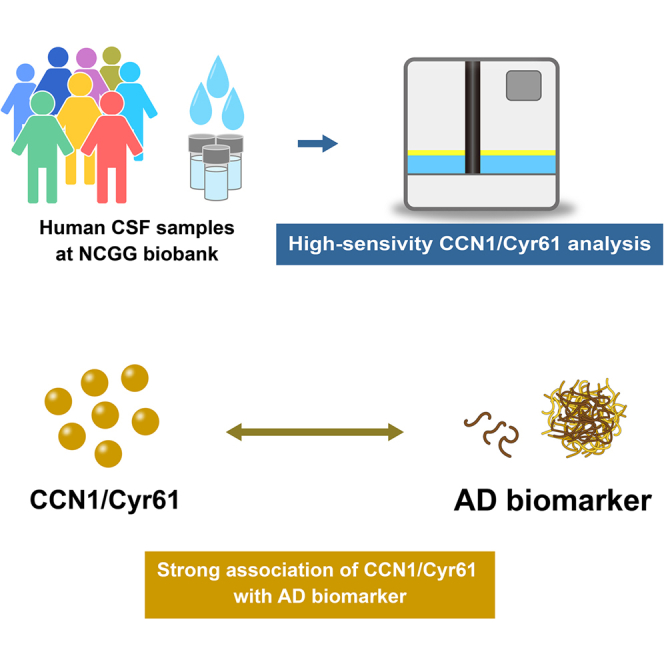

## Introduction

Developments of biomarkers for Alzheimer’s disease (AD), such as β-amyloid (Aβ), phosphorylated tau (phospho-tau or p-tau), and neurofilament L, have improved pathology-based diagnosis.^1^^,^^2^ In addition, developing biomarkers that reflect the pathological and biological processes is essential for understanding the mechanisms of the onset and progression of AD and for developing new therapeutic drugs. The strongest risk for AD is aging, and the number of cellular senescence cells increases with age.^3^^,^^4^ Previously, astrocytic senescence was reported in human AD,^5^ and recently, senescent microglial cells have been discovered in AD model mice,^6^ and it has been suggested that removing senescent cells using senolytic reagents may suppress the progression of neurodegenerative diseases including AD.^6^^,^^7^ However, it is not yet clear when and how cellular senescence contributes to AD, and no biomarkers related to cellular senescence have yet been reported.

CCN (cellular communication network factor) family member 1 (CCN1)/cysteine-rich angiogenic inducer 61 (Cyr61) is a member of the CCN family of matricellular proteins, which are involved diverse biological processes, including embryonic development, angiogenesis,^8^ wound healing,^9^ and inflammation.^10^ Moreover, CCN1/Cyr61 induces cellular senescence, which promotes wound healing and heart regeneration.^9^^,^^11^ In the brain, CCN1/Cyr61 has been shown to be induced by neuronal stimulation,^12^^,^^13^ astrocytic activation,^14^ and ischemic stroke,^15^ and influence neuroinflammation, blood-brain barrier integrity, and glial cell activity, and thus are expected to be involved in neurological diseases, including AD.^16^

Previously, we have reported a gene cluster, which includes CCN1/Cyr61, that is increased in AD mice model with obesity/diabetes,^17^^,^^18^ which may represent genes upregulated in patients’ brains.^17^ Indeed, in humans, CCN1/Cyr61 expression is higher in the entorhinal cortex and hippocampus in AD patients than in control.^17^^,^^19^ Recently, CCN1/Cyr61 expression has been also reported to increase in other AD mice model.^20^ Because CCN1/Cyr61 codes a secreted protein, it might be suitable as the fluid biomarkers for AD pathology, while there have been no reports of measurement of CCN1/Cyr61 in cerebrospinal fluid (CSF).

This study aimed to make it possible to measure CCN1/Cyr61 in CSF and to examine the correlation between CCN1/Cyr61 and various AD biomarkers in CSF. Using a highly sensitive SIMOA assay, we have enabled to measure CCN1/Cyr61 levels in the CSF of 79 subjects who collected by the CSF tap test. Interestingly, CCN1/Cyr61 showed good associations with Aβ40 levels, Aβ42 levels, Aβ42/40 ratio, and p-tau levels, which are somewhat comparable with associations among AD-specific biomarkers. CCN1/Cyr61 was also moderately associated with glial markers, including YKL-40, sTREM2, and CD163, and a lymphatic endothelial marker, LYVE-1. While these cellular markers were also associated with Aβ and p-tau, effects were much weaker than those of CCN1/Cyr61. Thus, associations between CCN1/Cyr61 and Aβ species or p-tau are more robust, indicating a close relationship between this senescence-inducing protein and AD pathology at the levels of fluid biomarkers.

## Results

### Development of highly sensitive CCN1/Cyr61 quantification assay by SIMOA

**Figure 1:**
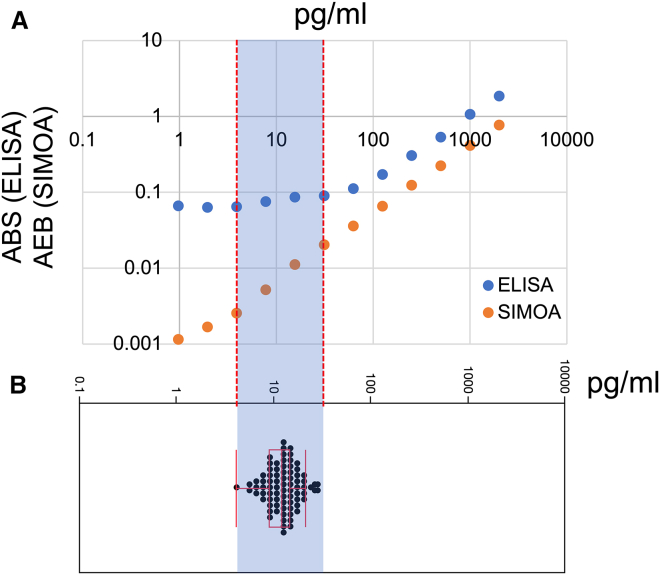
Development of highly sensitive SIMOA assay to detect CCN1/Cyr61 levels in CSF (A) Plots of ABS (absorbance value at 450 nm for ELISA, blue dots) or AEB (average number of enzymes per bead for SIMOA, orange dots) of serially diluted CCN1/Cyr61 standard protein in a representative assay. (B) CCN1/Cyr61 values in 3.8-fold diluted CSF samples from 78 subjects, measured by SIMOA assay, which falls within the dose-dependent range of serially diluted standard protein.

We initially attempted to measure CCN1/Cyr61 levels in CSF using a commercially available conventional ELISA kit with detection range of 31.2–2,000 pg/mL. However, we found that CCN1/Cyr61 levels in a pooled CSF sample were nearly below the detection limit, even when 30 μL of CSF sample (i.e., corresponding to a 3.3-fold dilution) was applied to the ELISA plate (data not shown). Therefore, we developed a highly sensitive SIMOA assay using the same combination of ELISA antibodies. The detection limit of the SIMOA assay became approximately 1 pg/mL, indicating a 30-fold increase in sensitivity compared to conventional ELISA (Figure 1A). This level of sensitivity was sufficient to quantify CCN1/Cyr61 even from 6 μL of pooled CSF sample, with the measured values falling within the linear range in a serial dilution experiment. Moreover, this SIMOA assay showed high specificity for CCN1, with no detectable cross-reactivity to other CCN family proteins (Figure S1). Thus, we proceeded to measure CCN1/Cyr61 levels in CSF from 79 subjects. After excluding one subject whose coefficient of variation exceeded 100% in duplicate assays, we calculated CCN1/Cyr61 values based on the standard curve. The minimum value, median value, and maximum values are 4.1, 12.1, and 26.3 pg/mL, respectively, all of which were in the range of the standard curve (Figure 1B). As these values were obtained from 26 μL of CSF sample (i.e., corresponding to a 3.8-fold dilution), we corrected for this dilution factor to obtain its concentration and then proceeded with analyses to evaluate changes in CCN1/Cyr61 levels within the cohort. We first analyzed the associations of CCN1 with age, sex, APOE4 genotype, and Mini-Mental State Examination (MMSE) scores. Indeed, CCN1/Cyr61 levels were positively associated with age (r = 0.33, *p* = 0.0029; Figure S2A). While there was a trend (but not significant) of reduction in CCN1/Cyr61 levels in females (Figure S2B), APOE4 did not affect CCN1/Cyr61 levels (Figure S2C). Of note, CCN1/Cyr61 was slightly but significantly elevated in clinically diagnosed AD (*p* = 0.0359; Figure S2D). In contrast, there was no association between CCN1/Cyr61 and MMSE (Figure S2E).

### Significant correlation of CCN1/Cyr61 levels with AD biomarkers

**Figure 2:**
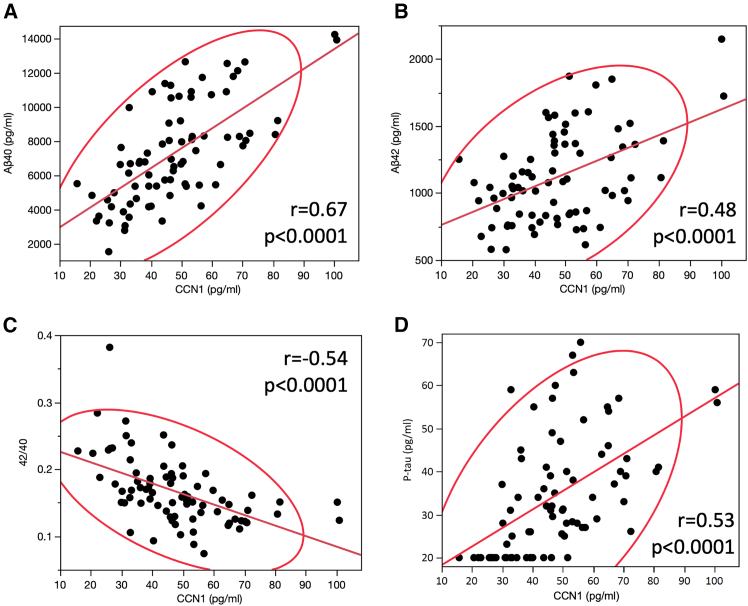
Correlation of CCN1/Cyr61 levels with AD biomarkers CCN1/Cyr61 levels are plotted against Aβ40 levels (A), Aβ42 levels (B), Aβ42/40 ratio (C), and p-tau levels (D) with a linear regression line and 95% confidence ellipse. Correlation coefficient (r) and *p* value were acquired by Pearson correlation test.

We then analyzed the relationship between CCN1/Cyr61 and AD biomarkers in CSF, including Aβ40, Aβ42, Aβ42/40 ratio, and phospho- and total-tau. Indeed, CCN1/Cyr61 showed good association with Aβ40 levels (r = 0.67, *p* < 0.0001; Figure 2A). While the correlation was relatively weak, CCN1/Cyr61 also showed significant association with Aβ42 levels (r = 0.48, *p* < 0.0001; Figure 2B). Moreover, CCN1/Cyr61 better associated with the Aβ42/40 ratio (r = −0.54, *p* < 0.0001; Figure 2C). Regarding tau, CCN1/Cyr61 showed good association with p-tau (r = 0.53, *p* < 0.0001; Figure 2D), and relatively weak but significant association with t-tau (r = 0.38, *p* = 0.0006). When adjusted for age, the associations between CCN1/Cyr61 and Aβ40, Aβ42, Aβ42/40 ratio, and p-tau remained statistically significant (Table S1). To further assess the relevance of these associations, we examined correlations among AD biomarkers. These associations, including those with CCN1, are presented in a scatterplot matrix (Figure S3). Aβ40 showed a strong association with Aβ42 (r = 0.78, *p* < 0.0001), and good associations with Aβ42/40 ratio (r = −0.68). p-tau was also strongly correlated with t-tau (r = 0.77), Aβ40 (r = 0.65), and Aβ42/Aβ40 ratio (r = −0.51). In contrast, t-tau showed weaker associations with Aβ40 (r = 0.41), Aβ42 (r = 0.10), and Aβ42/40 ratio (r = −0.51). Taken together, the strength of correlations between CCN1/Cyr61 and AD biomarkers, particularly with Aβ40 and p-tau, appears to be comparable to those between p-tau and Aβ species, supporting the potential relevance of CCN1/Cyr61 related with AD biomarkers.

### Correlation of CCN1/Cyr61 levels with glial or other cellular markers

**Figure 3:**
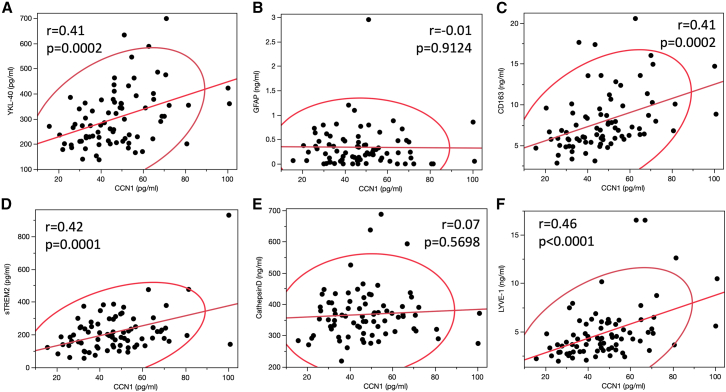
Correlation of CCN1/Cyr61 levels with glial markers CCN1/Cyr61 levels are plotted against YKL-40 levels (A), GFAP levels (B), CD163 levels (C), sTREM2 levels (D), Cathepsin-D levels (E), and LYVE-1 levels (F) with a linear regression line and 95% confidence ellipse. Correlation coefficient (r) and *p* value were acquired by Pearson correlation test.

We next evaluated the relationships between CCN1/Cyr61 and various glial markers, YKL-40, GFAP, CD163, sTREM2, and cathepsin D. CCN1/Cyr61 was significantly associated with YKL-40 levels (r = 0.41, *p* = 0.0002), but not with GFAP, another astrocytic marker (r = 0.01, *p* = 0.9124) (Figures 3A and 3B). CCN1/Cyr61 also showed significant associations with CD163, a macrophage/microglia marker (r = 0.41, *p* = 0.0002), and sTREM2, a microglia marker (r = 0.42, *p* = 0.0001) (Figures 3C and 3D). However, there was no significant association between CCN1/Cyr61 and cathepsin D, that is upregulated in disease-associated microglia^21^ (r = 0.07, *p* = 0.5698) (Figure 3E). In addition, we measured levels of LYVE-1, a lymphatic endothelial maker, and found a significant association with CCN1/Cyr61 (r = 0.46, *p* < 0.0001) (Figure 3F). To assess how CCN1/Cyr61 and these glial or other cellular marker relate to AD biomarkers, we summarized their associations in Table 1. Among these proteins, CCN1/Cyr61 consistently showed stronger associations with Aβ40, Aβ42, Aβ42/40 ratio, and p-tau, compared to those of these cellular markers. These results further support the potential relevance of CCN1/Cyr61 related with AD biomarkers.

**Table 1:** Correlation of CCN1/Cyr61 or glial or other cellular markers with AD biomarkers

	Aβ40		Aβ42		Aβ42/40		Phospho-tau		Total-tau	
	*r*	*p* Value	*r*	*p* Value	*r*	*p* Value	*r*	*p* Value	*r*	*p* Value
CCN1/Cyr61	0.67	<0.0001	0.48	<0.0001	−0.54	<0.0001	0.53	<0.0001	0.38	0.0006
YKL-40	0.34	0.0022	0.26	0.0207	−0.28	0.0132	0.42	0.0001	0.39	0.0004
GFAP	−0.05	0.6558	−0.05	0.6733	0.01	0.9438	0.03	0.9825	0.02	0.871
CD163	0.30	0.0070	0.14	0.2143	−0.34	0.0023	0.45	<0.0001	0.38	0.0005
sTREM2	0.35	0.0018	0.32	0.0038	−0.24	0.0303	0.39	0.0003	0.27	0.0154
Cathepsin D	0.10	0.3742	0.06	0.6069	−0.06	0.6212	−0.09	0.4327	−0.02	0.8827
LYVE-1	0.40	0.0003	0.32	0.0040	−0.27	0.0146	0.31	0.0051	0.21	0.0671

Correlation coefficient (r) and *p* value were acquired by Pearson correlation test.

## Discussion

The most notable finding of the current study is the strong association between CCN1/Cyr61 and Aβ species or p-tau. Previously reported biomarkers associated with Aβ species and/or p-tau include sTREM2, YKL-40, GFAP, t-tau, and Neurogranin.^22^^,^^23^^,^^24^^,^^25^ Importantly, CCN1/Cyr61 showed the strongest correlation with Aβ species and/or p-tau among the biomarkers examined in this study, including YKL-40, GFAP, CD163, sTREM2, cathepsin D, and LYVE-1. In particular, among AD-specific biomarkers (Aβ40, Aβ42, p-tau, and t-tau), the association between CCN1/Cyr61 and Aβ40 was the strongest. CCN1/Cyr61 is expressed in glial cells, especially astrocytes, or endothelial cells in the brain.^14^^,^^20^^,^^26^ Although the causal relationship between CCN1/Cyr61 and Aβ40 remains unsolved, CCN1/Cyr61 may be released from astrocytes and/or endothelial cells in response to Aβ accumulation. Alternatively, CCN1/Cyr61 might increase Aβ production or impair its clearance. Or, CCN1/Cyr61 and Aβ might be co-secreted by activated neurons, since both CCN1/Cyr61 and Aβ can be induced by neuronal stimulation.^12^^,^^13^ The source of CCN1/Cyr61 in CSF should be investigated in future studies.

Senescence involves a phenomenon called SASP (senescence-associated secretory phenotype), wherein senescent cells secrete cytokines, growth factors, matrix-remodeling enzymes, lipids, extracellular vesicles, and miRNAs, and exert pathophysiological function.^4^^,^^27^^,^^28^ Previously, astrocytic senescence was reported in human AD using p16ink4a and matrix metalloproteinase-1 (MMP-1) expression as markers of senescence.^5^ More recently, microglial senescence was reported in an AD model using high-throughput mass cytometry.^6^ However, the nature of cellular senescence in human AD remains unclear. Given that CCN1/Cyr61 is highly expressed in astrocytes and vascular endothelial cells,^14^^,^^20^^,^^26^ CCN1/Cyr61 secreted by these cells may induce senescence in microglia. As no single molecular marker is specific to cellular senescence,^4^ analysis of CCN1/Cyr61 together with other senescent biomarkers, including SASP-related molecules, may contribute to understanding of the role of Senescence in AD pathogenesis. Recently, CCN1/Cyr61 secreted from astrocytes has been reported to be involved in microglial phagocytosis of myelin debris^29^ and in maintaining neuronal networks.^30^ Measuring CCN1/Cyr61 in CSF may help clarify its roles in CNS pathophysiology.

AD CSF biomarkers might not be relied upon to differentiate iNPH from iNPH with comorbid AD,^31^ as the pathophysiology of iNPH is characterized by reduced periventricular metabolism.^32^^,^^33^^,^^34^ Nevertheless, the strong correlations observed between CCN1/Cyr61 and Aβ species or p-tau are unlikely to be coincidental. Their *p* values were below 0.0001, which would withstand correction for multiple comparisons, including Bonferroni adjustment (Table S2). Also, their associations are still significant even when adjusting for multiple confounders, including age, gender, APOE4 status, disease status, and glial or other cellular markers in CSF (Table 2). Also, as a pilot analysis, we additionally examined 21 CSF samples from patients with AD or MCI collected by conventional lumbar puncture for diagnostic purposes. Despite the limited sample size and the lack of healthy controls, CCN1/Cyr61 still showed significant associations with Aβ40 and p-tau, although the strengths of these associations were relatively weaker (Figure S4). Therefore, validation of the current findings in larger cohorts of conventionally collected CSF samples from patients and healthy controls will be important. To clarify the pathophysiological role of CCN1/Cyr61 in AD, we have developed conditional CCN1/Cyr61 knockout mice and are currently analyzing these mice crossed with AD models. We also performed preliminary immunohistochemical analyses of CCN1/Cyr61 in AD autopsy brains, which indicated that CCN1/Cyr61 colocalizes with Aβ in the cerebrovasculature and in senile plaques, suggesting the underlying mechanisms by which CCN1/Cyr61 associates with Aβ species and p-tau (Figure S5).

**Table 2:** Effects of CCN1/Cyr61 on AD biomarkers in multiple regression models

	Aβ40		Aβ42		Aβ42/40		Phospho-tau		Total-tau	
	Estimate	*p* Value	Estimate	*p* Value	Estimate	*p* Value	Estimate	*p* Value	Estimate	*p* Value
Model #1	116.1	<0.0001	9.57	<0.0001	−0.00157	<0.0001	0.430	<0.0001	3.06	0.0006
Model #2	108.5	<0.0001	9.02	<0.0001	−0.00149	<0.0001	0.421	<0.0001	3.15	0.0008
Model #3	107.5	<0.0001	8.56	0.0002	−0.00155	<0.0001	0.423	<0.0001	3.34	0.0006
Model #4	111.3	<0.0001	9.03	<0.0001	−0.00161	<0.0001	0.445	<0.0001	3.39	0.0005
Model #5	117.7	<0.0001	9.41	<0.0001	−0.00164	<0.0001	0.421	<0.0001	2.94	0.0022
Model #6	98.5	<0.0001	6.69	0.0097	−0.00157	<0.0001	0.361	0.0003	2.49	0.0136

Model #1 includes no covariate. Model #2 includes age as other covariates. Model #3 includes age and gender as other covariates. Model #4 includes age, gender, and APOE4 status as other covariates. Model #5 includes age, gender APOE4 status, and disease status (clinically diagnosed AD) as other covariates. Model #6 includes age, gender APOE4 status, disease status, and other glial or other cellular markers in CSF as other covariates.

In conclusion, CCN1/Cyr61 associates with Aβ species and p-tau levels in CSF. The association is considerably stronger than those observed with typical glial or other cellular markers, suggesting the potential role for CCN1/Cyr61 in AD-related molecular pathogenesis. Further studies analyzing larger cohorts, including definitive AD patients, and additional senescence-related markers would validate and extend the current findings.

### Limitations of the study

This study has several limitations. First, the CSF samples used in the primary analyses were collected through the CSF tap test from individuals with suspected iNPH. Although this relatively uniform clinical background reduces heterogeneity, it also limits the generalizability of the findings. Second, although we found no association between CCN1/Cyr61 and MMSE score, longitudinal studies are needed to assess whether CCN1/Cyr61 can predict cognitive decline in AD. Third, the underlying mechanisms by which CCN1/Cyr61 associates with Aβ species and p-tau remain still unclear. Further analysis is needed to elucidate the pathophysiological mechanisms underlying the relationship between CCN1/Cyr61 and Aβ and p-tau.

## Resource availability

### Lead contact

Requests for further information and resources should be directed to and will be fulfilled by lead contact, Naoyuki Sato (nsato@ncgg.go.jp).

### Materials availability

This study generated high sensitive quantification assay for CCN1/Cyr61, whose protocols and reagents are available from the lead contact upon request.

### Data and code availability

• All data generated or analyzed during this study are available from the lead contact upon request.
• No code was used in this study.
• Requests for other data or information pertaining to this article should be directed to the lead contact.

## Acknowledgments

This work was supported in part by the Research Funding for Longevity Sciences from the 10.13039/501100007312National Center for Geriatrics and Gerontology (19-3, 21-12, and 24-16 to N.S.; 22-13 and 25-15 to H.M.); Grants-in-Aid from Japan Promotion of Science (MEXT26293167, MEXT15K15272, MEXT17H04154, MEXT21H02844, and MEXT24K02361 to N.S.); a 10.13039/100007449Takeda Science Foundation Research Encouragement Grant (to M.S.); 10.13039/100009619Japan Agency for Medical Research and Development (JP24wm0425019 and JP24dk0207074h0001 to Y.S.); 10.13039/501100001691Japan Society for the Promotion of Science 10.13039/501100001691KAKENHI (JP 22H04923 to Y.S.); Integrated Research Initiative for Living Well with Dementia of the 10.13039/100030953Tokyo Metropolitan Institute for Geriatrics and Gerontology to Y.S.; 10.13039/501100003478Ministry of Health, Labour and Welfare Research on rare and intractable diseases (JPMH23FC1008 to Y.S.). We would like to thank the laboratory members in the Department of Aging Neurobiology and staffs in the memory clinic and the Biobank at National Center for Geriatrics and Gerontology and Dr. Masahiko Bundo for collecting CSF samples. We also thank Prof. Guojun Bu for the antibodies and Prof. Masashi Narita and Prof. Akinori Nakamura for the helpful discussion.

## Author contributions

M.S., H.M., and N.S. contributed to the concept and study design; M.S., H.M., T.S., C.T., K.K., G.G., K.K., M.M., Y.S., A.S., F.Y., T.I., A.F., and N.S. contributed to data acquisition and analysis; M.S., H.M., and N.S. contributed to drafting the manuscript and figures. All the authors edited and reviewed the final manuscript.

## Declaration of interests

The authors declare no competing interests.

## STAR★Methods

### Key resources table

**Table:** 

REAGENT or RESOURCE	SOURCE	IDENTIFIER
**Antibodies**		

Anti-Human Amyloidβ (35–40)	IBL	1A10; RRID: AB_3697111
Anti-Human Amyloidβ (x-42)	Mayo Clinic	MM26–2.1.3; RRID: AB_11212601
Anti-Human Amyloidβ (17–24)	Biolegend	4G8; RRID: AB_2734548
Anti-GFAP antibody (capture)	US Biological	G2032-27J; RRID: AB_2247496
Anti-GFAP antibody (detection)	Abcam	ab79203; RRID: AB_2109662
Anti-cathepsin-D antibody (capture)	US Biological	125465; RRID: AB_3751261
Anti-cathepsin-D antibody (detection)	R&D	AF1014; RRID: AB_2087218

**Biological samples**		

Human CSF samples	National Center for Geriatrics and Gerontology Biobank	https://www.ncgg.go.jp/research/lab/cfa/laboratory/biobank/home.html

**Chemicals, peptides, and recombinant proteins**		

Homebrew assay development kit	Quanterix	101354
CCN2 recombinant protein	R&D	9190-CC-050
CCN3 recombinant protein	R&D	1640-NV-050
GFAP recombinant protein	Abnova	H00002670-P01
Cathepsin-D recombinant protein	R&D	1014-AS-010
Horseradish peroxidase (HRP)-linked Avidin-D	Vector Laboratories	A-2004
3,3′,5,5′-tetramethylbenzidine	Sigma-Aldrich	T2885
Amyloidβ 1-42	AnaSpec	AS-20276
Amyloidβ 1-40	Peptide Institute	4367-v
Albumin, from Bovine Serum, pH5.2	FUJIFILM Wako Pure Chemical Corporation	015–21274
Tris-Buffered Saline (TBS) Tablets, pH7.6	TaKaRa	T9141

**Critical commercial assays**		

Cyr61/CCN1 DuoSet ELISA kit	R&D	DY4055
Pierce^TM^ EDC	ThermoFisher Scientific	A35391
Human Chitinase 3-like 1 (YKL-40) DuoSet ELISA kit	R&D	DY2599
sTREM2 DuoSet ELISA kit	R&D	DY1828-05
LYVE-1 DuoSet ELISA kit	R&D	DY2089
Human CD163 DuoSet ELISA kit	R&D	DY1607-05

**Software and algorithms**		

JMP Pro	SAS	version 13.0.0

**Other**		

MaxiSorp^TM^ immunoassay plates	ThermoFisher Scientific	442404
Polypropylene microplates	ThermoFisher Scientific	249944
SIMOA HD-X	Quanterix	Software3.1
iMark plate reader	BIO-RAD	13451

### Experimental model and study participant details

#### Participants and human CSF samples

This study included CSF samples from 79 Japanese subjects who underwent a CSF tap test due to suspected idiopathic normal pressure hydrocephalus (iNPH) (mean age = 77.7 years (range, 63–94), male: female = 48:31). Between 2016 and 2021, CSF was collected and stored in an onsite biobank. The lumbar puncture was performed by using a 19G spinal needle by neurosurgeons, and approximately 30 mL of CSF was drained by gravity drip. The first 10 mL drained CSF was used for clinical testing and retained for an onsite biobank. CSF samples were temporarily stored at 4°C, then centrifuged at 2,350 ×g at 4°C for 10 min, dispensed in 500 μL aliquots into 1.5 mL polypropylene tubes (ProteosaveSS, Sumitomo Bakelite, Japan), and stored at −80°C. The diagnosis of Alzheimer’s disease was determined clinically based on the patient’s medical history, medication, typical clinical symptoms, and brain imaging findings. After considering the results of CSF tap test and the shunt surgery, 24 patients were finally diagnosed with Alzheimer’s disease at the time of lumber puncture.

#### Ethical approval

All procedures performed in this study were approved by the institutional ethics committee at the National Center for Geriatrics and Gerontology (approval number 1608–2) and conducted in accordance with the Declaration of Helsinki. The human biological samples with provided informed consent were used under the approval of institutional ethics committee and the onsite biobank.

### Method details

#### CCN1/Cyr61 measurement

The levels of CCN1/Cyr61 were first tried to be measured by human Cyr61/CCN1 DuoSet ELISA kit (#DY4055, R&D, Minneapolis, MN, USA) using MaxiSorp™ immunoassay plates (#442404, ThermoFisher Scientific, Waltham, MA, USA). To develop a high-sensitive CCN1/Cyr61 quantification assay using SIMOA (single molecule array), we customized this ELISA kit by using the Homebrew assay development kit, according to the manufacturer’s instruction (#101354, Quanterix, Billerica, MA, USA). In brief, ELISA coating antibodies were conjugated with carboxylated paramagnetic beads by using Pierce™ EDC (#A35391; ThermoFisher Scientific, Waltham, MA, USA), and used as capture reagents by diluting 1:58 in bead diluent buffer. CSF samples and CCN1 standard protein were diluted at an appropriate amount in TBS buffer with 0.2% bovine serum albumin, which was the same procedure as ELISA, and applied in polypropylene microplates (#249944, ThermoFisher Scientific) for SIMOA sample measurement. ELISA detection antibody and streptavidin-β-galactosidase (SBG) were prediluted 1:180 and 1:1000, respectively, in appropriate reagent buffers. All incubations and washing steps were performed on the fully automated SIMOA HD-X analyzer (Quanterix). First, 100 μL of prediluted sample was incubated with 25 μL of capture reagent for 15 min, followed by an automatic washing, and further incubation with 100 μL of prediluted detector antibody for 5 min and 15 s. After an automatic washing, the prediluted SBG reagent and its substrate (resorufin-β-D-galactopyranoside, RGP) were incubated for 5 min and 15 s was performed. After an automatic washing, the beads were transferred to the SIMOA discs, and signals were detected and counted. To confirm the specificity among CCN family members, recombinant proteins of CCN2 (#9190-CC-050, R&D) and CCN3 (1640-NV-050, R&D) were analyzed by the same assay.

#### Measurement of other proteins

The levels of Aβ42 (Aβ_x-42_) and Aβ40 (Aβ_x-40_) were determined by ELISAs as previously described with some modifications.^35^^,^^36^ The current ELISAs used end-specific monoclonal antibodies for capture (1A10 (IBL, Japan) for Aβ_x-40_ and MM26–2.1.3 (gifts from Dr. Guojun Bu at Mayo Clinic, USA) for Aβ_x-42_), a biotin-conjugated 4G8 antibody for detection (epitope: 17–24 a.a. of Aβ, Biolegend, San Diego, CA, USA) and each specific Aβ peptide for standards (Aβ1-42: #AS-20276, AnaSpec, Fremont, CA, and Aβ1-40: #4367-v, Peptide Institute, Osaka, Japan). The levels of phospho-tau (p-tau) 181 and total-tau (t-tau) were routinely determined at our hospital by each specific ELISA, which followed standard clinical methods to diagnose AD and Creutzfeldt–Jakob disease.^37^^,^^38^ Because twenty-one individuals had p-tau values below the detection limit (20 pg/mL), we conservatively assigned a value of 20 pg/mL to these samples. As a sensitivity analysis, we also observed similar results after removing these samples (data not shown). The levels of glial fibrillary acidic protein (GFAP) were determined by ELISA, as previously described,^36^ using rabbit polyclonal antibody for capture (#G2032-27J, US Biological, Salem, MA, USA), a biotin-conjugated mouse monoclonal GA5 antibody (#ab79203, Abcam, Cambridge, UK), and a standard recombinant protein (#H00002670-P01, Abnova, Taipei City, Taiwan). The levels of YKL-40, sTREM2, CD163, and LYVE-1 were determined by commercially available ELISA kits, according to the company’s instructions (R&D). The levels of cathepsin-D were determined by ELISA using rabbit polyclonal antibody for capture (#125465, US Biological), a biotin-conjugated goat polyclonal antibody (#AF1014, R&D), and a standard recombinant protein (#1014-AS-010, R&D). To detect these proteins in CSF, we applied samples with appropriate dilution, which were beforehand determined by a serial dilution experiment confirming linear reactivity depending on sample dilution (data not shown). Colorimetric quantification was performed using an iMark plate reader (BIO-RAD, Hercules, CA, USA) after incubations with horseradish peroxidase (HRP)-linked Avidin-D (Vector Laboratories, Burlingame, CA, USA), followed by incubation with 3,3′,5,5′-tetramethylbenzidine substrate (#T2885, Sigma-Aldrich, St. Louis, MO, USA).

### Quantification and statistical analysis

All measurements were performed in duplicate, and the average value was used. If duplicate values showed significant discrepancies, the measurements were either reanalyzed or excluded from the analysis. In addition to standard correlation analyses, the associations between CCN1/Cyr61 and AD biomarkers were examined using multivariable linear regression models adjusted for age, gender, APOE4 status, disease status (clinically diagnosed AD), and glial or other cellular markers measured in CSF. All the statistical analyses were performed by JMP Pro (version 13.0.0; SAS, Cary, NC, USA). *p*-values of less than 0.05 were considered significant.
