# Supplementary material for: CCN1/Cyr61 associates with β-amyloid levels in human cerebrospinal fluids
Source: iScience. 2026 Jun 5;29(6):116244. doi: 10.1016/j.isci.2026.116244 (PMC13265892; doi:10.1016/j.isci.2026.116244)
Supplement: Document S1. Figures S1–S5 and Tables S1 and S2 [file mmc1.pdf]

iScience, Volume *29*

Supplemental information

CCN1/Cyr61 associates with β-amyloid levels in human cerebrospinal fluids

Mitsuru Shinohara, Hiroyuki Momota, Tsuyoshi Saito, Chisako Takenobu, Kensaku Kasuga, Ghupurjan Gheni, Kaori Kawai, Maho Morishima, Yuko Saito, Akihiro Shindo, Fumihiko Yasuno, Takeshi Ikeuchi, Akio Fukumori, and Naoyuki Sato

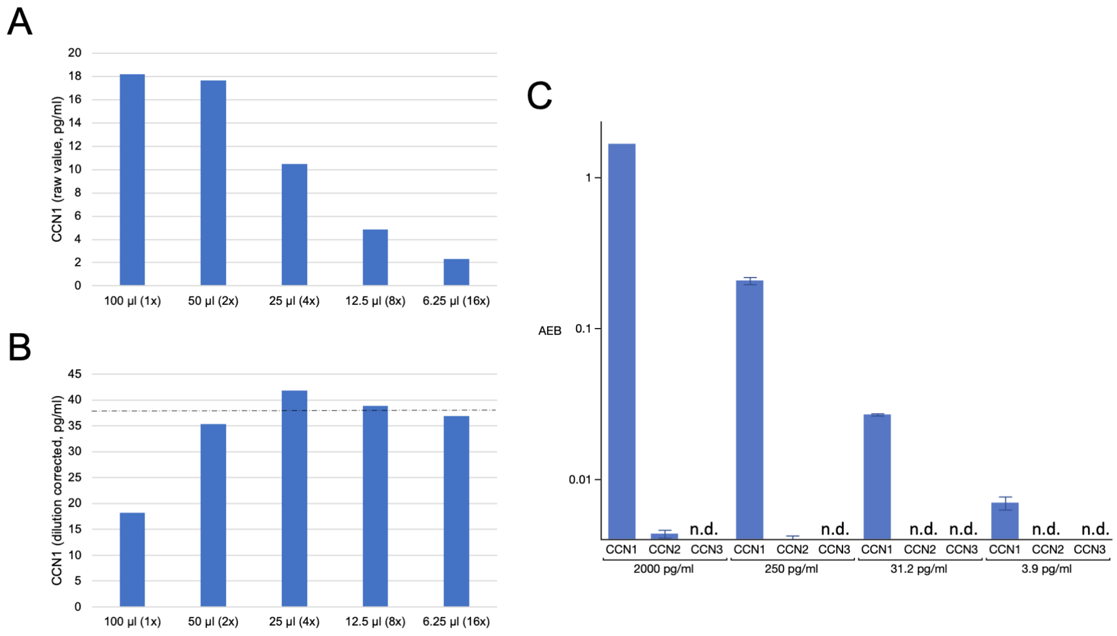

Supplementary Figure 1. Validation of CCN1 SIMOA assay. (A) Raw CCN1/Cyr61 concentrations, and (B) dilution-corrected CCN1/Cyr61 concentrations measured by serial dilution of a pooled CSF sample. The dashed line shows the mean of dilution-corrected CCN1/Cyr61 levels among 2-fold (50 µl, 2x), 4-fold (25 µl, 4x), 8-fold (12.5 µl, 8x), and 16-fold (6.25 µl, 16x) diluted samples, suggesting consistent CCN1/Cyr61 measurements across this range of dilutions. (C) Average number of enzymes per bead (AEB) for recombinant CCN1, CCN2, and CCN3 proteins at the tested concentrations. n.d., not detected (below background AEB).

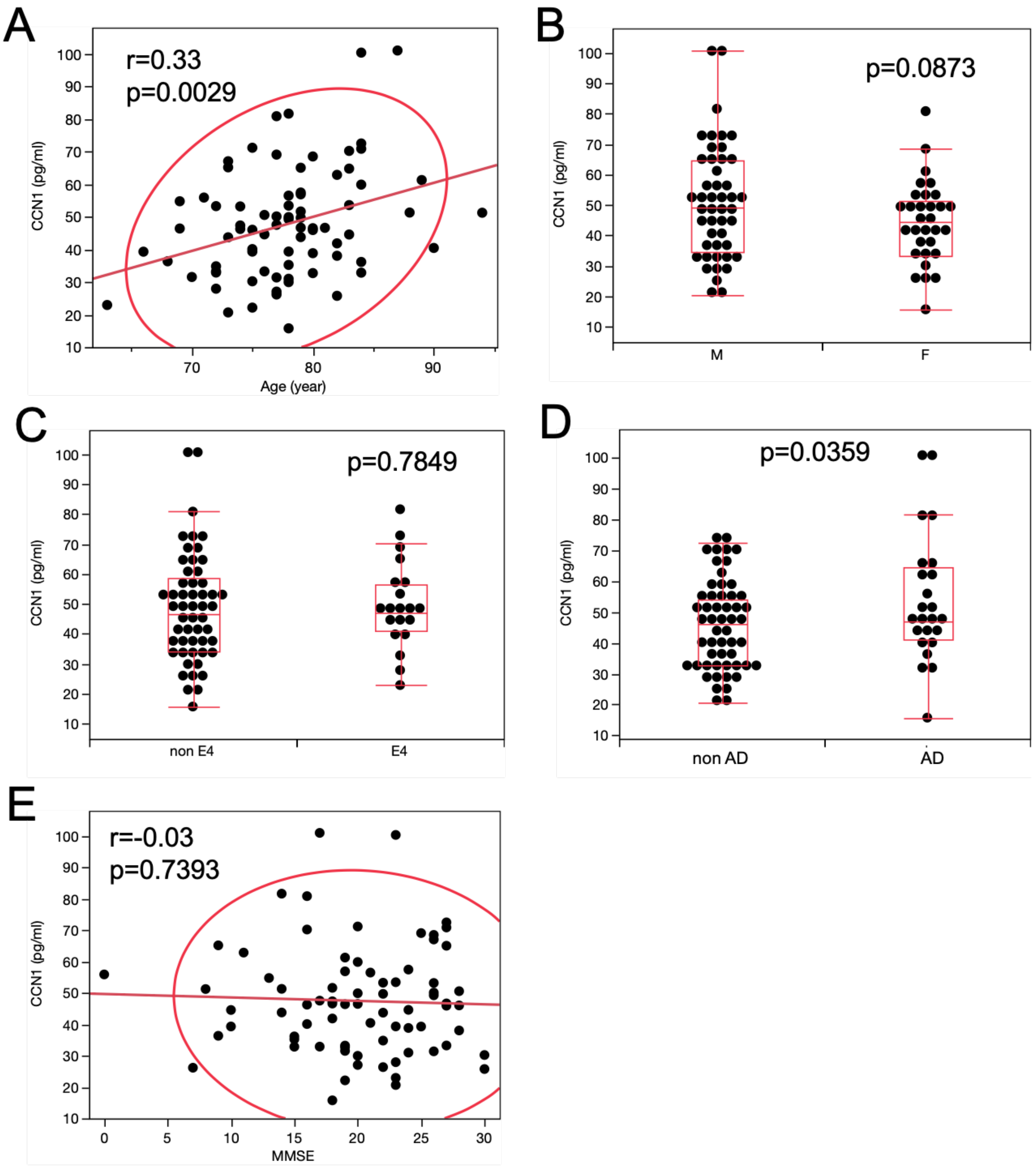

Supplementary Figure 2. Basic information of CCN1/Cyr61 distribution in CSF by age, sex, APOE4, AD and MMSE. (A) CCN1/Cyr61 levels are plotted against age with a linear regression line and 95% confidence ellipse. (B) CCN1/Cyr61 levels in males and females are compared and plotted with a box-and-whisker diagram. (C) CCN1/Cyr61 levels in non-E4 carriers and E4 carriers are compared and plotted with a box-and-whisker diagram. (D) CCN1/Cyr61 levels in clinically-diagnosed AD patients and non-AD subjects are compared and plotted with a box-and-whisker diagram. (E) CCN1/Cyr61 levels are plotted against MMSE score with a linear regression line and 95% confidence ellipse. (B, C, D) P-values were acquired by Student's t-test. (A, E) Correlation coefficient (r) and P-value were acquired by Pearson correlation test.

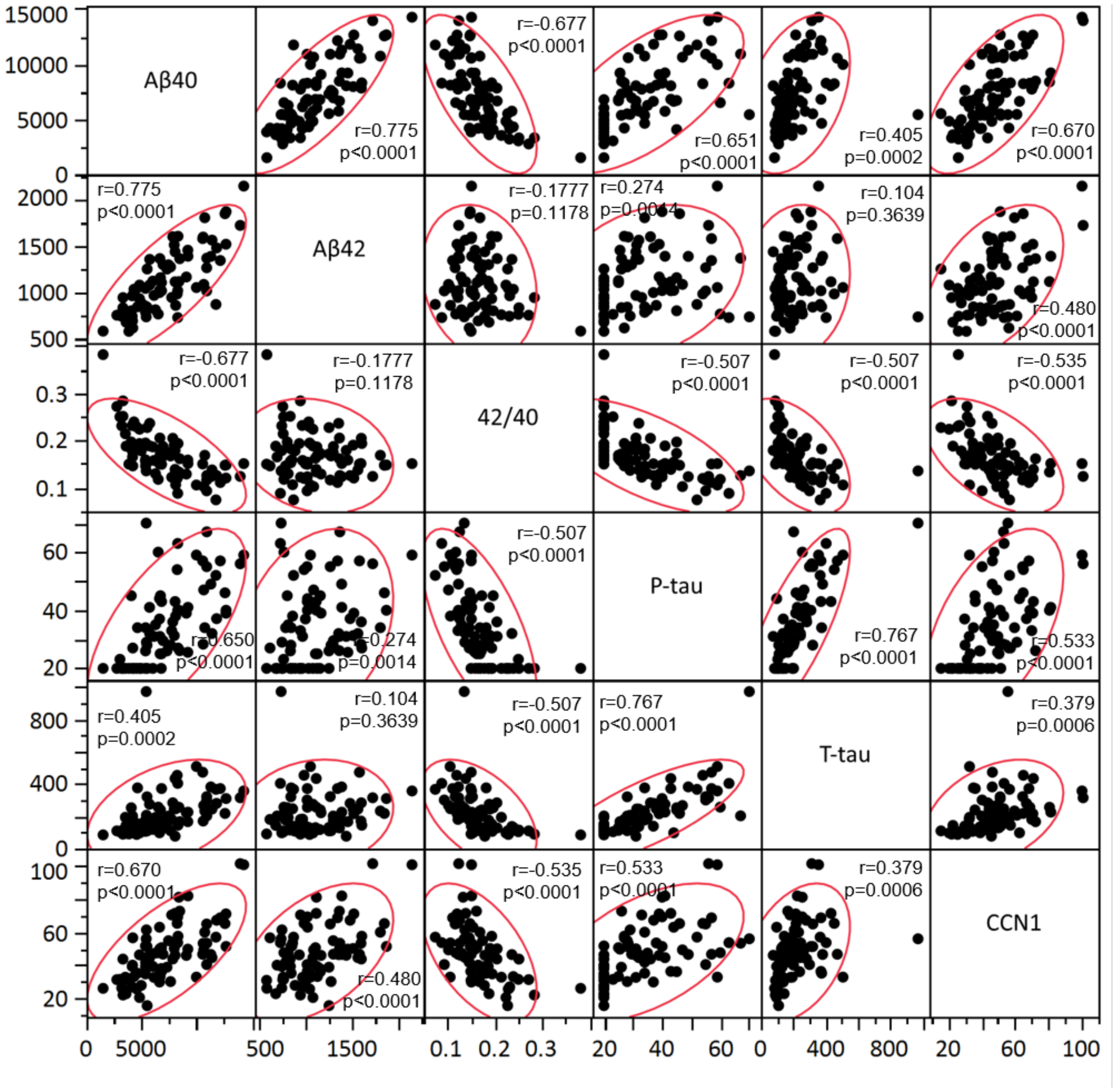

Supplementary Figure 3. Scatterplot matrix showing correlations among AD biomarkers and CCN1/Cyr61 with 95% confidence eclipse, correlation coefficient (r) and p-values, which were acquired by Pearson correlation test.

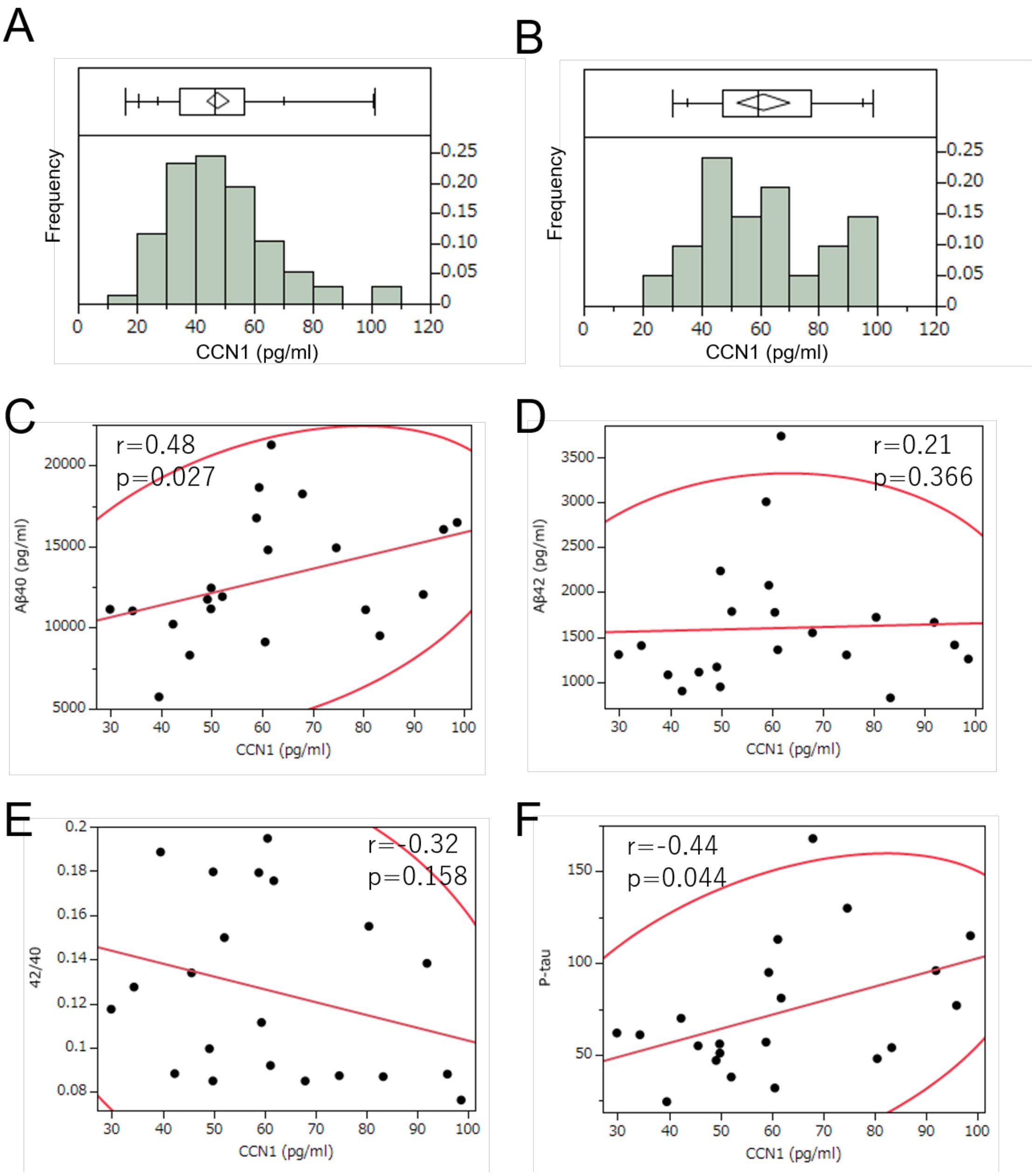

Supplementary Figure 4. Analysis of additional samples of 21 AD or MCI patients collected by conventional lumbar puncture for AD diagnostic purpose. Histograms showing distribution of CCN1/Cyr61 levels in the original samples (79 subjects, A) and these samples (21 subjects, B). CCN1/Cyr61 levels in 21 subjects are plotted against Aβ40 levels (C), Aβ42 levels (D), Aβ42/40 ratio (E), and p-tau levels (F) with a linear regression line and 95% confidence ellipse. Correlation coefficient (r) and P-value were acquired by Spearman's correlation test.

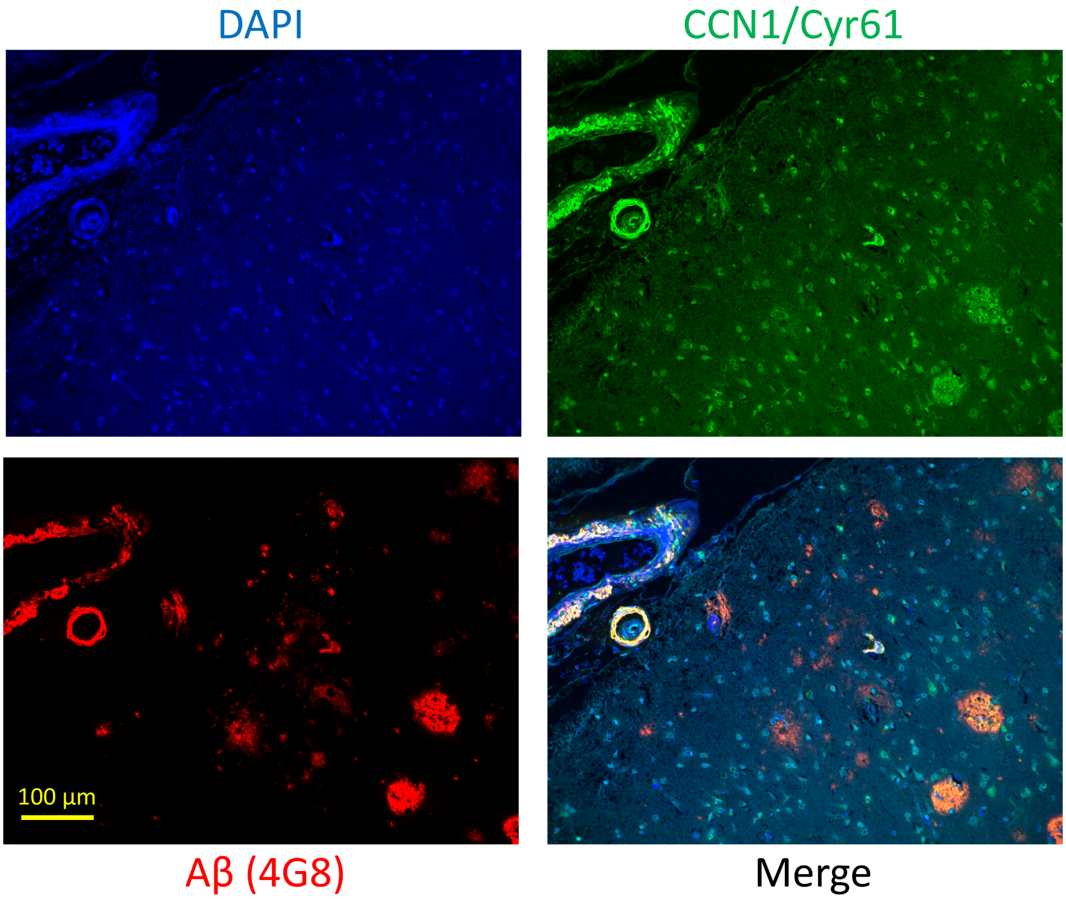

Supplementary Figure 5. Fluorescent staining of CCN1/Cyr61 and Aβ pathologies. A brain section of the dorsolateral frontal cortex of an AD case (male, 83 years) was triple-stained using DAPI (blue; #H1800, Vector Labs), an anti-CCN1/Cyr61 antibody (green; #NB100-356, Novus Biologicals), and an anti-Aβ antibody (red; clone 4G8, epitope: amino acids 17-24 of Aβ; BioLegend). Individual channel images and a merged image are shown. The scale bar represents 100 µm.

Supplementary Table 1. Correlation of CCN1/Cyr61 or glial or other cellular markers with AD biomarkers adjusted for age

	**Aβ40**		**Aβ42**		**Aβ42/40**		**Phospho-tau**		**Total-tau**	
	Partial r	***P* Value**	***Partial r***	***P* Value**	***Partial r***	***P* Value**	***Partial r***	***P* Value**	***Partial r***	***P* Value**
**CCN1/Cyr61**	0.67	<0.0001	0.48	<0.0001	-0.54	<0.0001	0.51	<0.0001	0.38	0.0006
**YKL-40**	0.34	0.0022	0.26	0.0207	-0.28	0.0132	0.40	0.0003	0.39	0.0004
**GFAP**	-0.05	0.6558	-0.05	0.6733	0.01	0.9438	-0.05	0.6560	0.02	0.871
**CD163**	0.30	0.0070	0.14	0.2143	-0.34	0.0023	0.43	0.0001	0.38	0.0005
**sTREM2**	0.35	0.0018	0.32	0.0038	-0.24	0.0303	0.36	0.0008	0.27	0.0154
**Cathepsin D**	0.10	0.3742	0.06	0.6069	-0.06	0.6212	-0.06	0.5844	-0.02	0.8827
**LYVE-1**	0.40	0.0003	0.32	0.0040	-0.27	0.0146	0.30	0.0085	0.21	0.0671

Partial correlation coefficients and P-value were calculated after controlling for age.

Supplementary Table 2. Correlation of CCN1/Cyr61 or glial or other cellular markers with AD biomarkers

	**Aβ40**			**Aβ42**			**Aβ42/40**			**Phospho-tau**			**Total-tau**		
	r	***P* Value**	***P'* Value**	***r***	***P* Value**	***P'* Value**	***r***	***P* Value**	***P'* Value**	***r***	***P* Value**	***P'* Value**	***r***	***P* Value**	***P'* Value**
**CCN1/Cyr61**	0.67	<0.0001	<0.0001	0.48	<0.0001	0.0003	-0.54	<0.0001	<0.0001	0.53	<0.0001	<0.0001	0.38	0.0006	0.0195
**YKL-40**	0.34	0.0022	0.0771	0.26	0.0207	0.7252	-0.28	0.0132	0.4615	0.42	0.0001	0.0048	0.39	0.0004	0.0137
**GFAP**	-0.05	0.6558	1.0000	-0.05	0.6733	1.0000	0.01	0.9438	1.0000	0.03	0.9825	1.0000	0.02	0.871	1.0000
**CD163**	0.30	0.0070	0.2443	0.14	0.2143	1.0000	-0.34	0.0023	0.0807	0.45	<0.0001	0.0009	0.38	0.0005	0.0187
**sTREM2**	0.35	0.0018	0.0625	0.32	0.0038	0.1329	-0.24	0.0303	1.0000	0.39	0.0003	0.0121	0.27	0.0154	0.5400
**Cathepsin D**	0.10	0.3742	1.0000	0.06	0.6069	1.0000	-0.06	0.6212	1.0000	-0.09	0.4327	1.0000	-0.02	0.8827	1.0000
**LYVE-1**	0.40	0.0003	0.0088	0.32	0.0040	0.1413	-0.27	0.0146	0.5114	0.31	0.0051	0.1777	0.21	0.0671	1.0000

Correlation coefficient (r) and P-value were acquired by Pearson correlation test. Adjusted p-values (P'-value) were calculated using the Bonferroni correction, by multiplying the original p-values by the number of correlation analyses (n = 35).
